# *Candida albicans* biofilm development is governed by cooperative attachment and adhesion maintenance proteins

**DOI:** 10.1038/s41522-019-0094-5

**Published:** 2019-08-23

**Authors:** Andrew D. McCall, Ruvini U. Pathirana, Aditi Prabhakar, Paul J. Cullen, Mira Edgerton

**Affiliations:** 1 Department of Oral Biology, School of Dental Medicine, The State University of New York at Buffalo, Buffalo, NY 14214 USA; 2 Department of Biological Sciences, College of Arts and Sciences, The State University of New York at Buffalo, Buffalo, NY 14214 USA

**Keywords:** Biofilms, Pathogens

## Abstract

The opportunistic fungal pathogen *Candida albicans* is capable of adhering to the oral mucosa despite forces created by salivary flow. Although many fungal adhesion proteins have been identified, less is known about the temporal development of cell adhesion and biofilm growth in a flow environment. In this study, we use a flow system with real-time imaging of *C. albicans* cells as they adhere and grow. Rates of cell attachment and dispersion of *C. albicans* knockout strains of putative adhesins, transcription factors, and deletions with a hyperfilamentous phenotype were quantified during 18 h of biofilm development. Cell adhesion under flow is a multi-phase process initiated with cell rolling, then an initial firm attachment to the substrate occurs. After attachment, cells enter a growth phase where cells either commit to adherence or disperse. *C. albicans* Δ*eap1*, Δ*hwp2*, Δ*hyr1*, and Δ*ihd1* cells had significantly reduced initial attachment and subsequent adhesion, while Δ*als1*/Δ*als3* had no change in initial attachment but reduced adhesion maintenance. WT cells had increased adhesion during the late growth phase when hyphae were more highly expressed. Hyperfilamentous strains had 10-fold higher total biofilm growth, a result of significantly reduced detachment rates, showing that hyphal morphogenesis is important for adhesion maintenance in the developing biofilm. The rate of *C. albicans* biomass dispersion was most important for determining the density of the mature biomass. Adhesion maintenance was mediated in part by Ywp1, a protein previously thought to regulate dispersion, thus it functions as an adhesion maintenance protein in *C. albicans*.

## Introduction

*Candida albicans* is an opportunistic fungal pathogen and the main causative agent of oropharyngeal candidiasis.^1^ Cells from oral infections may be dispersed through salivary flow to the gut, and then disseminated through the blood stream to the liver, kidney and brain.^2,3^ We recently developed a flow system of biofilm development that introduces the effects of shear force representing estimates of saliva flow over mucosal surfaces.^4^ In addition, this imaging system enables quantification of *C. albicans* adhesion and biofilm growth in real-time.^5^ This technique has allowed us to calculate early cellular attachment to surfaces, as well as less-well defined later events including maintenance of cellular attachment (adhesion maintenance) and the loss of adhesive contacts resulting in detachment and dispersion of fungal cells. However, the identity of critical fungal surface adhesins that contribute to adhesion maintenance and dispersion is not known.

Despite the critical roles that biofilms play in the attachment and penetration of fungal cells into host tissues, many basic features of biofilm development and organization remain mysterious. For example, early adhesive steps in biofilm formation appear to be mechanistically distinct from later events supporting growth of cells in flow. The key regulators of biofilm development in these two phases are not understood. The best characterized surface adhesin proteins include members of the *ALS* (agglutinin-like sequence) gene family^6^ (encoding Als1-9), as well as Eap1 and Hwp1. Als1 and Als3 have high sequence similarity and functional redundancy and both interact with Hwp1 to facilitate cell-to-cell adhesion.^7^ *C. albicans* Eap1 participates in adhesion to polystyrene substrates, as demonstrated by the increase in surface adhesion of *Saccharomyces cerevisiae* cells in which *C. albicans EAP1* was expressed.^8^ Other *C. albicans* cell-wall proteins have been shown to play direct or indirect roles in adhesion to human cell lines or in cellular aggregation, including Hwp2, Pga1, and members of the Sap family.^9–11^

Our flow experiments allowed us to separate biofilm development into adhesion and growth phases in order to measure the initial rate of *C. albicans* cell attachment independently from maturation of the biofilm biomass. Surprisingly, we found that the rate of biomass detachment (governed by cell adhesion maintenance forces) was most important for growth of the mature biomass, and that initial cell attachment rates did not always predict total biomass. Therefore, we expected that temporal expression of *C. albicans* surface adhesins would be responsible for overall biofilm adhesion maintenance. To begin identification of these adhesins, we compared *C. albicans* adhesin knockout mutants to WT cells and found that *C. albicans HYR1*, *EAP1*, *HWP2*, and *IHD1* all contribute to both initial cell attachment and adhesion maintenance, while *ALS1/ALS3* adhesins are mainly involved in initial attachment. Surprisingly, we found that some *C. albicans* strains previously identified as adhesion deficient (Δ*efg1* and Δ*bcr1*) did not differ in initial adhesion but were defective for adhesion maintenance. In addition, we discovered that Ywp1 appears to be partly responsible for maintaining adhesion to the substratum during the growth phase, and thus we assign its function as adhesion maintenance. Understanding the process of adhesion and release in *C. albicans* biofilms could help target specific fungal proteins crucial to adhesion maintenance in order to reduce biofilm formation by this opportunistic pathogen.

## Results

### Quantification *C. albicans* biofilm growth

Previously, we found that biofilms formed at 23 °C with flow grew evenly over the substrate permitting accurate real-time quantitation of adhesion and dispersion, while those grown at 37 °C formed dense microcolonies separated by large gaps that did not allow reproducible quantitation.^4^ As our assay requires imaging at a single location before any cells are present and for the full 18 h experiment, data was acquired at 23 °C rather than 37 °C for more accurate quantitation. Furthermore, cells were incubated in the recirculating flask at 37 °C, thus inducing expression of hyphal genes prior to adhesion.

We mechanistically defined two phases of biofilm growth as the Attachment phase (0–2 h) and Growth phase (2–18 h) since they represent two conditions within our flow system. During the Attachment phase, all *C. albicans* cells (including detached cells) were recirculated over the substratum for 2 h. The attachment phase consisted of both cells that adhered to the surface, as well as cells that attached to other cells adherent to the surface (cell–cell adhesion). While we are able to quantitate cell–cell adhesion independently of cell-surface adhesion, we found no significant differences in cell-cell adhesion of any of the tested strains. Therefore, we combined both of these events for analyses of the Attachment phase.

For Growth phase analyses, all *C. albicans* cells that detached from the surface or biofilm were removed by filters, so that no new cells were added to the media flowing over the biofilm surface. Therefore, biofilm growth during the Growth phase occurred only from cells that remained attached to the surface. Keeping the media cell-free allowed us to analyze the effect of adhesion maintenance on biofilm growth and development under flow, and removed dispersed cells (allowing calculation of detachment rates) from those that remained adherent.

**Fig. 1:**
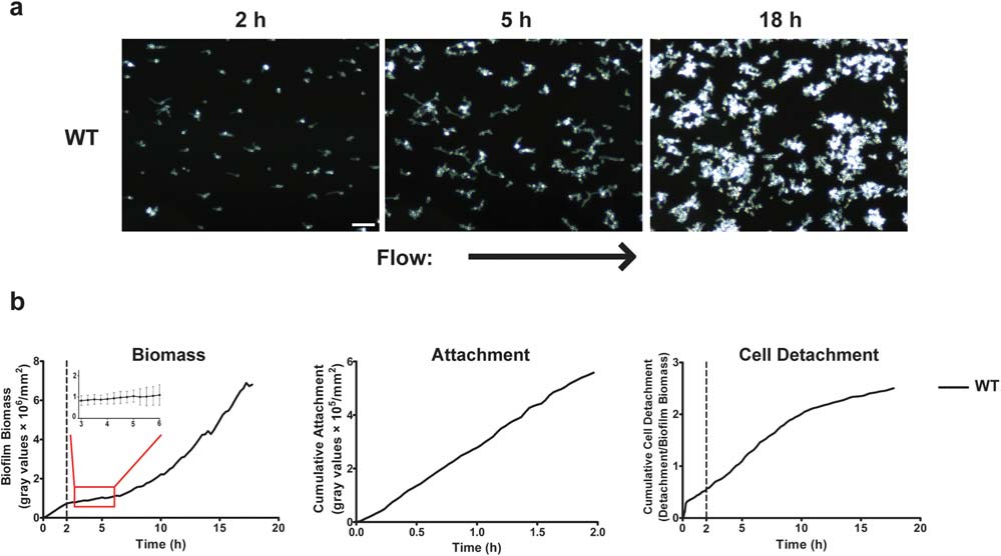
Adhesion and growth of wild-type cells under flow. **a** Representative dark field images of biofilm formation under flow are shown for wild-type CAI4 + *URA* cells grown at 23 °C at 2, 5, and 18 h of growth. Arrow indicates direction of flow for every image. **b** the total biomass within the imaging region (determined by densitometry analysis), the rate of cell attachment, and the biomass detachment (detachment rate normalized to the biomass) over time are shown. Scale bar indicates 50 µm. Data are means of *n* ≥ 3 independent experiments. Inset shows means ± s.d.

Wild-type (WT) CAI4 + *URA* cells were used for comparison with isogenic mutants, although we also examined *C. albicans SC5314*, and *SN250* in the flow system and found both strains to exhibit quantitatively similar biofilm growth parameters. *C. albicans* WT cells first rolled over the surface then adhered predominantly as yeast cells with occasional pseudohyphal attachment during the attachment phase (Fig. 1 and Supplementary Movie 1). A relatively uniform coating of clustered yeast cells adhered to the substratum, with cell clusters ranging in size from 1–2 cells to ~30 cells (Fig. 1a). The larger cell clusters were the result of cell–cell adhesion events, which, on average, accounted for half of the adhesion events between 1–2 h. The cumulative biofilm biomass (Fig. 1b, Biomass), attachment rate (Fig. 1b, Attachment), and detachment rate (Table 1) for WT cells for the 2 h attachment phase was calculated.

**Table 1:** Quantification of *Candida albicans* adhesion under flow

	2 h Biofilm biomass^a^ (×10^5^)	Attachment rate^b^ (×10^5^)	CDR 0–2 h^c^
WT	7.18 (1.65)	2.98 (0.01)	0.21 (0.01)
Δ/Δ*als1/3*	2.63 (0.75)	1.18 (0.02)	0.40 (0.02)
Δ*hyr1*	0.82 (0.54)	0.42 (0.01)	0.41 (0.02)
Δ*hwp2*	0.24 (0.29)	0.25 (0.01)	0.58 (0.03)
Δ*eap1*	0.78 (0.23)	0.34 (0.03)	0.13 (0.01)
Δ*ihd1*	0.32 (0.27)	0.21 (<0.01)	0.40 (0.02)
WT	7.18 (1.65)	2.98 (0.01)	0.21 (0.01)
Δ*hog1*	4.20 (1.99)^NS^	1.71 (0.03)	0.19 (0.01)^NS^
Δ*sfl1*	8.00 (2.16)^NS^	1.70 (0.06)	0.05 (<0.01)
WT	7.18 (1.65)	2.98 (0.01)	0.21 (0.01)
Δ*bcr1*	7.19 (4.25)^NS^	3.22 (0.08)	0.47 (0.03)
Δ*efg1*	6.38 (4.42)^NS^	1.57 (0.02)	0.12 (0.01)
Rapamycin	2.41 (0.87)^NS^	1.17 (0.03)	0.21 (0.01)^NS^
WT	7.18 (1.65)	2.98 (0.01)	0.21 (0.01)
Δ*ywp1*	8.27 (2.54)^NS^	1.74 (0.05)	0.07 (<0.01)

Each value represents a mean or best-fit slope of *n* ≥ 3 experiments, with standard deviation in parentheses

NS indicates no significant difference between each strain compared to wild-type CAI4 cells. All other values were significant (*p* < 0.05)

^a^Biofilm Biomass was determined by full frame densitometry analysis at 2 h, with values given in gray values/mm^2^

^b^Attachment Rate represents the average biomass of newly attached cells/mm^2^/h within the first 2 h (the attachment phase). Cell detachment does not impact the attachment rate

^c^Cell Detachment Rate (CDR) was calculated as the average total detachment rate (average biomass of newly detached cells/mm^2^/h) divided by the biomass of biofilm for the first two hours. Values indicate the average proportion of biomass that detaches from the biofilm per hour

We observed that biomass formation during the Growth phase of WT cells occurred at different rates between 2–5 h and from 5–18 h (Fig. 1). Therefore, we further defined two stages of biofilm growth to better measure these rates by linear regression analyses as early biofilm growth rate (BGR) (2–5 h) and late BGR (5–18 h, Table 2). For WT cells, late BGR was found to be more than double the early BGR (Table 2, and Fig. 1b). Since detachment of cells is critical for development of the total biomass during the Growth phase, we calculated the cell detachment rate (CDR) and its inverse Adhesion Maintenance Strength (AMS) (Table 3) for the Growth phase. Interestingly, for WT cells, the early CDR was much higher than late CDR, indicating that the strength of adhesion (AMS) increased with the developing biomass in late growth stage. This further indicates that AMS positively correlates with the ability to both maintain and gain biomass over time. We next examined these parameters to understand how specific fungal proteins and regulatory elements contribute to biofilm development.

**Table 2:** The rate of growth over time in *Candida albicans* biofilms grown under flow

	Early BGR^a^ (×10^5^)	Late BGR^a^ (×10^5^)	Difference in BGR (Late–Early) (×10^5^)
WT	2.30 (0.06)	4.87 (0.37)	2.57
Δ/Δ*als1/3*	1.00 (0.03)	0.41 (0.05)	−0.59
Δ*hyr1*	0.28 (0.02)	0.15 (0.03)	−0.13
Δ*hwp2*	0.05 (0.01)	0.01 (0.00)	−0.04
Δ*eap1*	0.14 (0.02)	0.00 (0.00)	−0.14
Δ*ihd1*	0.05 (0.01)	−0.00 (0.00)	−0.05
WT	2.30 (0.06)	4.87 (0.37)	2.57
Δ*hog1*	2.31 (0.11)^NS^	43.85 (3.36)	41.54
Δ*sfl1*	4.08 (0.11)	55.09 (0.87)	51.01
WT	2.30 (0.06)	4.87 (0.37)	2.57
Δ*bcr1*	1.94 (0.17)^NS^	−0.30 (0.04)	−2.24
Δ*efg1*	1.72 (0.15)	−0.63 (0.07)	−2.35
Rapamycin	0.79 (0.04)	0.68 (0.02)	−0.11
WT	2.30 (0.06)	4.87 (0.37)	2.57
Δ*ywp1*	4.01 (0.11)	−1.19 (0.20)	−5.20

Each value represents a best-fit slope of *n* ≥ 3 experiments, with standard deviation in parentheses. Early and Late values are determined from data collected between 0 to 5 h and 5 to 18 h, respectively

NS indicates no significant difference between each strain compared to wild-type CAI4 cells. All other values were significant (*p* < 0.05)

^a^Biofilm Growth Rate (BGR) was calculated as the best fit slope of the biomass over time in gray values/mm^2^/h obtained by linear regression

**Table 3:** Detachment rate and adhesion maintenance of *Candida albicans* biofilms under flow

	Early CDR^a^	Late CDR^a^	Early AMS^b^	Early AMS log_2_ (fold change) over WT	Late AMS^c^	Late AMS log_2_ (fold change) over WT
WT	0.18 (0.01)	0.10 (<0.01)	5.45		9.88	
Δ/Δ*als1/3*	0.22 (0.01)^NS^	0.17 (<0.01)	4.58^NS^	NS	5.97	−0.73
Δ*hyr1*	0.23 (0.01)	0.36 (0.01)	4.33	−0.33	2.81	−1.81
Δ*hwp2*	0.34 (0.02)	0.20 (0.01)	2.98	−0.87	5.08	−0.96
Δ*eap1*	0.27 (0.01)	0.28 (0.01)	3.74	−0.54	3.54	−1.48
Δ*ihd1*	0.38 (0.01)	0.13 (<0.01)	2.60	−1.07	7.49	−0.40
WT	0.18 (0.01)	0.10 (<0.01)	5.45		9.88	
Δ*hog1*	0.11 (0.01)	0.03 (<0.01)	9.23	0.76	36.76	1.90
Δ*sfl1*	0.04 (<0.01)	0.04 (<0.01)	24.51	2.17	23.20	1.23
WT	0.18 (0.01)	0.10 (<0.01)	5.45		9.88	
Δ*bcr1*	0.41 (0.01)	0.21 (<0.01)	2.47	−1.14	4.71	−1.07
Δ*efg1*	0.20 (0.01)^NS^	0.20 (<0.01)	5.06^NS^	NS	4.98	−0.99
Rapamycin	0.13 (0.01)	0.12 (<0.01)	7.70	0.50	8.22	−0.27
WT	0.18 (0.01)	0.10 (<0.01)	5.45		9.88	
Δ*ywp1*	0.10 (<0.01)	0.33 (<0.01)	10.40	0.93	3.06	−1.69

Each value represents a best-fit slope of *n* ≥ 3 experiments, with standard deviation in parentheses. Data was divided into Early (0–5 h) and Late (5–18 h) phases, as many strains displayed an apparent change in rate around 5 h

NS indicates no significant difference between each strain compared to wild-type CAI4 cells. All other values were significant (*p* < 0.05)

^a^Cell Detachment Rate (CDR) was calculated as the average total detachment rate (average biomass of newly detached cells/mm^2^/h) divided by the biomass of biofilm. Values indicate the average proportion of biomass that detaches from the biofilm per hour

^b^Adhesion Maintenance Strength (AMS) was calculated as the inverse of the BDR. Value is reflective of the strength with which a cell remains adhered to the cells or substrate of a biofilm over time

### *C. albicans* adhesins contribute to biofilm growth through initial attachment

**Fig. 2:**
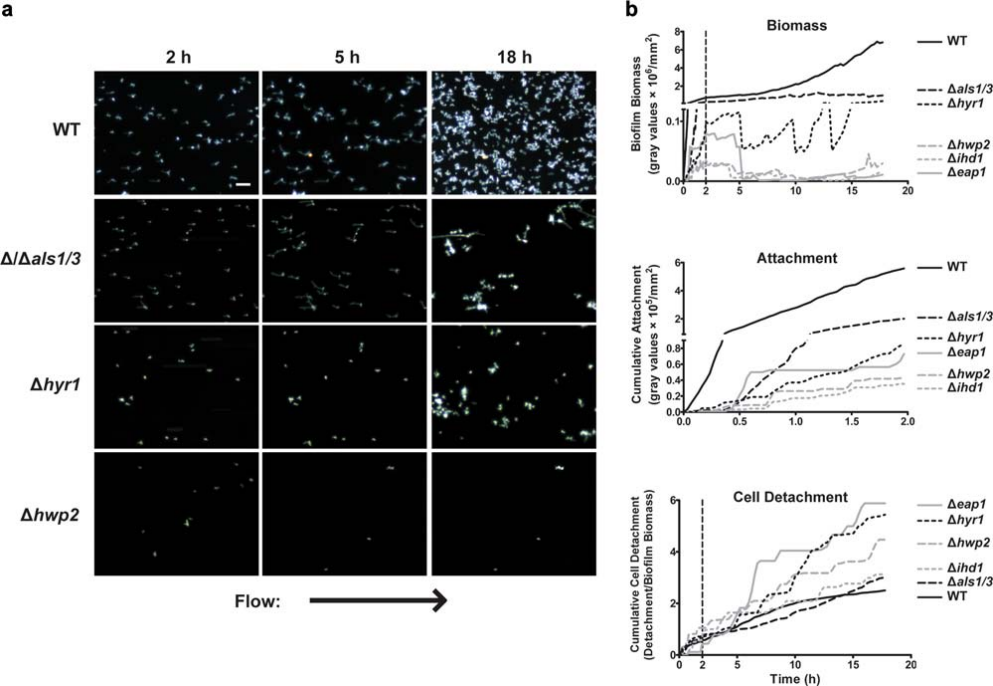
Adhesin knockouts have reduced initial rates of attachment under flow. **a** Representative dark field images of biofilm formation under flow are shown for CAI4 + *URA*, Δ*als1*/Δ*als3*, Δ*hyr1*, Δ*eap1*, Δ*ihd1*, and Δ*hwp2* cells at 2, 5, and 18 h of growth at 23 °C. Arrow indicates direction of flow for every image. **b** the total biomass within the imaging region (determined by densitometry analysis), the rate of cell attachment, and the biomass detachment (detachment rate normalized to the biomass) over time are shown for each strain. Scale bar indicates 50 µm. Data are means of *n* ≥ 3 independent experiments

Since many *C. albicans* adhesins are presumed to be important for biofilm development, we analyzed several known and putative *C. albicans* adhesins using knockout strains including Δ*eap1*, Δ*hwp2*, Δ*hyr1*, Δ*ihd1*, and an Δ*als1*/Δ*als3* double deletion mutant (Fig. 2a, Supplementary Movies 2–4). Since *ALS1* and *ALS3* have a substantial overlap in the function,^7^ we used a *C. albicans* strain with deletion of both genes. All of the selected *C. albicans* adhesin knockouts showed a marked reduction in total biofilm biomass (at 18 h) with *C. albicans* Δ*hwp2*, Δ*eap1*, and Δ*ihd1* strains showing the most severe reduction (Fig. 2, Table 1). *C. albicans* Δ*hwp2*, Δ*eap1*, and Δ*ihd1* strains had a ten-fold reduction in attachment rate and a two-fold increase in dispersion rate during the attachment phase (Table 1). These cells also had a higher CDR (Fig. 2b) and a reduction in AMS (Table 3) resulting in nearly complete loss of total biofilm production (Fig. 2a). Although we expected that the *C. albicans* Δ*als1*/Δ*als3* strain would have the most severe defect in adhesion, these cells had only half the attachment rate and twice the dispersion rate resulting in 2.7-fold biomass reduction (2 h) compared to WT cells. By comparison, other adhesin mutants (specifically the Δ*hwp2* and Δ*ihd1* mutants) had 15-fold reduced 2 h biomass mainly due to a 10-fold reduction in attachment rates. Since we calculated that dispersion rate values above 0.36 contribute to loss of biomass, the detachment rates of *C. albicans* Δ*als1*/Δ*als3*, Δ*hwp2*, Δ*hyr1*, and Δ*ihd1* were just slightly above this threshold, showing that the main reason for reduction in biomass (at 2 h) for these mutants was defective attachment rates. Interestingly, while Δ*eap1* cells showed a reduced attachment rate similar to Δ*hwp2* and Δ*ihd1* mutants, they had higher biofilm biomass as a result of their lower dispersion rate (0.13 compared to 0.21 for WT) (Table 1).

We expected that the adhesion defects might be carried over to the Growth phase, and indeed found that the BGR in both early and late stages for *C. albicans* Δ*eap1*, Δ*hwp2*, Δ*hyr1*, Δ*ihd1* cells were significantly reduced compared to WT (Table 2). However, when we measured these cells’ adhesion strength during the Growth phase, we unexpectedly found only slightly higher detachment rates, so that AMS values were reduced by only 0.33–1.07 log_2_ fold (early growth) and 0.4–1.8 log_2_ fold (late growth) (Table 3). This suggested that the low 18 h biofilm biomass for *C. albicans* Δ*eap1*, Δ*hwp2*, Δ*hyr1*, and Δ*ihd1* cells was more a result of initial attachment defects, rather than loss of adhesion maintenance during the growth phase. Interestingly, *C. albicans* Δ*als1*/Δ*als3* cells had no statistically significant reduction in AMS in the early Growth phase, and only a −0.73 log_2_ fold change compared to WT in late Growth Phase (Table 3). Thus, the reduced 2 h attachment rate of *C. albicans* Δ*als1*/Δ*als3* cells was most crucial for its diminished final biofilm biomass, since these cells had small defects in AMS only during late Growth phase. The results further implied that different sets of *C. albicans* adhesins are responsible for initial attachment and for adhesion maintenance, and that they are likely to function cooperatively as shown by the substantial reduction in biofilm growth by deletion of just one of the tested adhesins.

### Adherence is promoted by hyphal formation

**Fig. 3:**
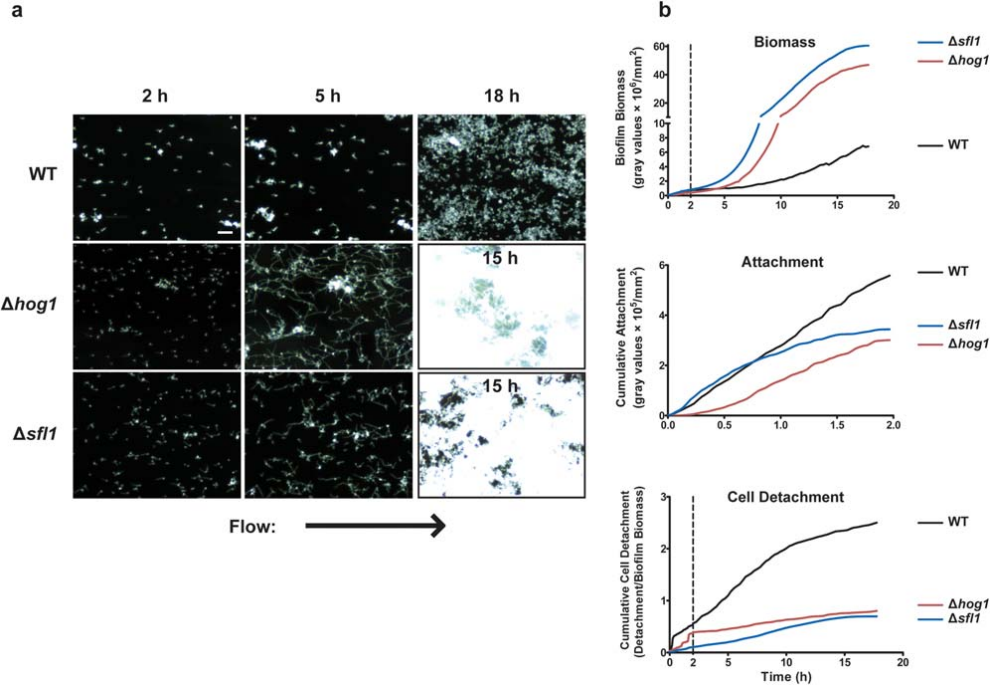
The hyperfilamentous mutants Δ*hog1* and Δ*sfl1* have greater adhesion and biofilm biomass. **a** Representative dark field images of biofilm formation under flow are shown for CAI4 + *URA*, Δ*hog1*, and Δ*sfl1* cells at 2, 5 and 18 h of growth at 23 °C. Arrow indicates direction of flow for every image. By 18 h of growth, the Δ*sfl1* strain had completely saturated our camera for every experiment, resulting in entirely white images. **b** The total biomass within the imaging region (determined by densitometry analysis), the rate of cell attachment, and the biomass detachment (detachment rate normalized to the biomass) over time are shown for each strain. Scale bar indicates 50 µm. Data are means of *n* ≥ 3 independent experiments

Many *C. albicans* adhesins are differentially expressed during hyphal morphogenesis.^12^ Since our evidence pointed to a role for multiple co-operative *C. albicans* adhesins, and WT cells had increased AMS during the late Growth phase when hyphae were more highly expressed, we expected that there might be a positive relationship between biofilm growth and the production of hyphae. To examine this relationship, we analyzed biofilm growth using two hyperfilamentous *C. albicans* mutants, Δ*hog1*^13^ and Δ*sfl1*.^14^ As anticipated, both of these strains were hyperfilamentous in our flow system (percentage of cells that had formed hyphae by 4 h were 92.3 and 91.6% for Δ*hog1* and Δ*sfl1*, respectively, compared to 4.0% for WT), and also were the two strains producing the highest biofilm biomass by 18 h (Fig. 3 and Supplementary Movies 5-6). During the initial 2 h Attachment phase, *C. albicans* Δ*hog1* and Δ*sfl1* cells had small immature hyphae and produced statistically equal biomasses as WT cells. During the early Growth phase in which hyphae matured, *C. albicans* hyperfilamentous strains had equivalent (Δ*hog1*) or 2-fold higher (Δ*sfl1*) biofilm growth compared to WT cells, while late biofilm growth rates for both Δ*hog1* and Δ*sfl1* strains (accompanied by robust hyphal formation) showed nearly a 10-fold increase compared to WT cells (Table 2). This was a result of the dramatically increased AMS of these strains which resulted in significantly reduced detachment rates during the Growth phase (Table 3 and Fig. 3). These results show that hyphal morphogenesis increases adhesion maintenance in the developing biofilm.

### Adhesion is maintained through an *EFG1* and *BCR1* regulated mechanism

**Fig. 4:**
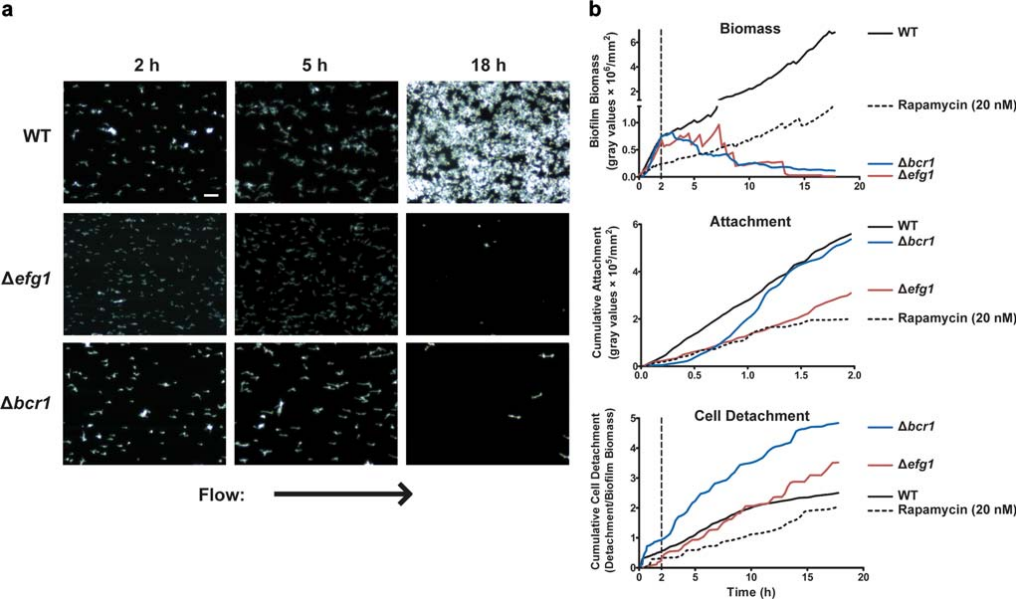
The transcription factor knockouts Δ*efg1* and Δ*bcr1* show early attachment, but do not remain adherent during growth. **a** Representative dark field images of biofilm formation under flow are shown for CAI4 + *URA*, Δ*efg1*, and Δ*bcr1* cells, as well as CAI4 cells treated with rapamycin (20 nM) at 2, 5, and 18 h of growth at 23 °C. Arrow indicates direction of flow for every image. **b** the total biomass within the imaging region (determined by densitometry analysis), the rate of cell attachment, and the biomass detachment (detachment rate normalized to the biomass) over time are shown for each strain and condition. Scale bar indicates 50 µm. Data are means of *n* ≥ 3 independent experiments

We further screened two *C. albicans* deletion mutants of transcription factors *EFG1* and *BCR1*, both known to be important for biofilm formation. Since *C. albicans* Efg1 and Bcr1 are key regulators of several adhesin genes, including most of the *ALS* gene family*, EAP1* and *HWP1*,^8,15–18^ as well as for static biofilm formation^16^ we expected that these knock-out mutants would also have defective attachment rates and/or increased detachment rates. The *C. albicans* Δ*efg1* strain was able to filament as pseudohyphae in the flow system, while the *C. albicans* Δ*bcr1* strain produced morphologically typical hyphae. Surprisingly, we noticed striking differences between Δ*efg1* and Δ*bcr1* compared to the single adhesin knockouts (Fig. 4, Supplementary Movies 7–8). Neither Δ*efg1* or Δ*bcr1* strains had defects in biofilm biomass at 2 h. *C. albicans* Δ*bcr1* cells had a similar attachment rate compared to WT cells, while *C. albicans* Δ*efg1* had a 50% reduction in attachment rate compared to WT cells (Fig. 4b and Table 1). However, *C. albicans* Δ*bcr1* cells had twice the detachment rate of WT cells, while *C. albicans* Δ*efg1* cells had half the detachment rate of WT (Table 1). Thus, Δ*efg1* or Δ*bcr1* strains had opposite phenotypes during the initial adhesion phase. In the early Growth phase, *C. albicans* Δ*efg1* cells had a statistically similar growth rate and detachment rate as WT cells, however this growth rate plummeted in the late growth phase due to a significant decrease in AMS (Table 3). In contrast, *C. albicans* Δ*bcr1* cells that had low detachment in the adhesion phase had significantly higher biomass detachment in both early and late Growth, as well as significantly reduced AMS. This reduction in AMS during the late growth phase of *C. albicans* Δ*bcr1* cells was so large (−1.14 to −1.07 log_2_ fold over WT) that by 18 h this strain had little to no remaining adherent biofilm cells. These temporal differences in the growth phase of AMS values between Δ*efg1* or Δ*bcr1* strains suggested that each strain expressed adhesion maintenance proteins at different times within the growth phases and at reduced levels. Furthermore, *C. albicans* Δ*bcr1* cells had robust adhesion to the substrate during the attachment phase, suggesting that adhesin gene expression (perhaps including *ALS1*, *ALS3*, *HWP2*, *HYR1*, and *IHR1*) was not altered during the first 2 h of growth.

It has previously been shown that sublethal doses of the fungistatic drug rapamycin results in an upregulation Als1, Als3 and Hwp1 by relieving Tor1 mediated repression of *EFG1* and *BCR1*.^15^ Thus, we measured the attachment and biofilm growth of WT *C. albicans* cells in the presence of 20 nM rapamycin, expecting that rapamycin treatment would improve initial attachment or increase adhesion maintenance strength through increased expression of these adhesin proteins (Fig. 4 and Supplementary Movie 9). Although the attachment rate for rapamycin-treated WT cells at 2 h was less than half that of untreated WT cells, rapamycin-treated cells had no difference in biomass detachment rate during the adhesion phase (Table 1). As expected, both early and late BGR was reduced due to drug inhibition (Table 2), however the AMS was significantly increased compared to untreated WT cells in the early growth phase and only slightly reduced during late growth phase (Table 3). Thus, rapamycin treatment of cells resulted in significantly higher AMS independent from their attachment rate, suggesting that additional factors, such as increased matrix formation as a result of upregulation of essential matrix formation genes (*KRE6*, *XOG1*, *SCW11*) by rapamycin,^15^ might play a role.

### Ywp1 is an adhesion maintenance protein during late biofilm growth

**Fig. 5:**
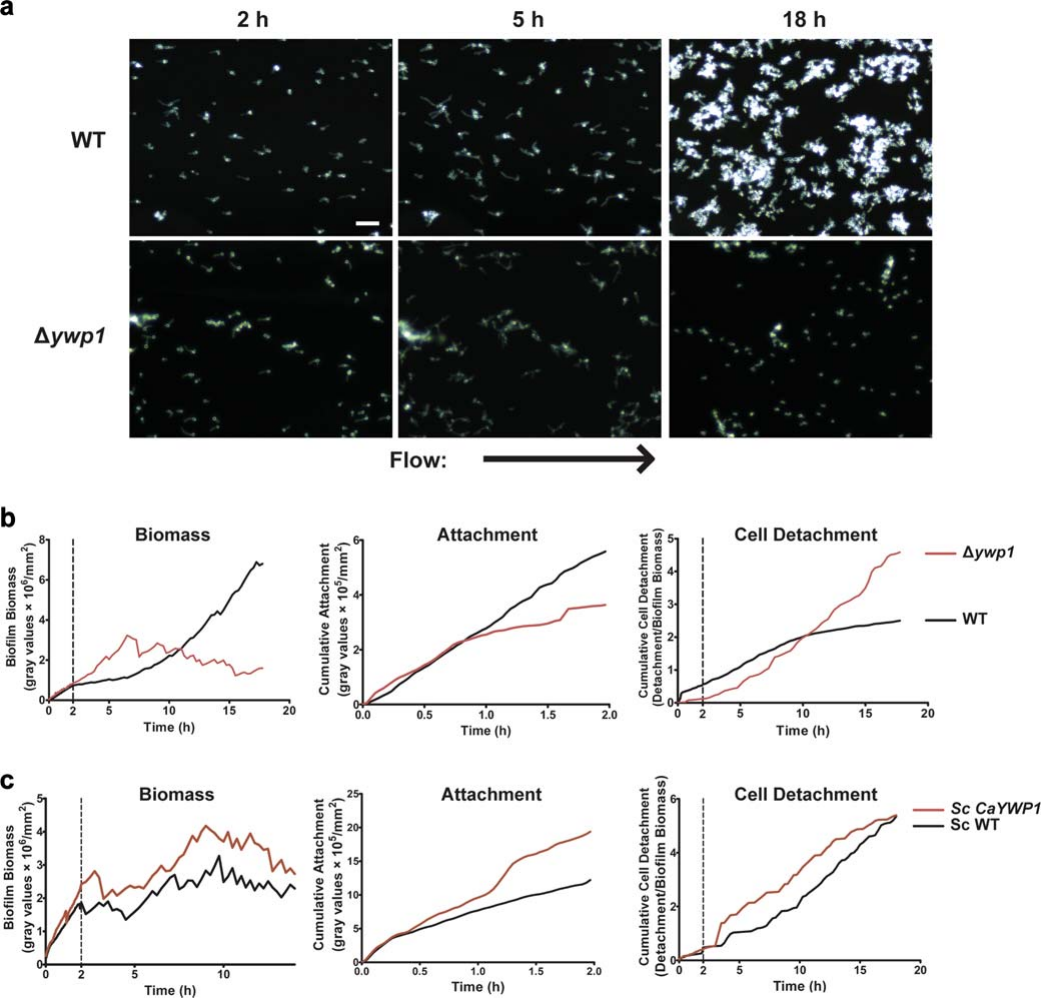
Adhesion maintenance is regulated at the cell wall through Δ*ywp1*. **a** Representative dark field images of biofilm formation under flow are shown for CAI4 + *URA*, and Δ*ywp1* cells at 2, 5, and 18 h of growth at 23 °C. Arrow indicates direction of flow for every image. **b** the total biomass within the imaging region (determined by densitometry analysis), the rate of cell attachment, and the biomass detachment (detachment rate normalized to the biomass) over time are shown for each *Candida albicans* strain. **c** the total biomass within the imaging region (determined by densitometry analysis), the rate of cell attachment, and the biomass detachment (detachment rate normalized to the biomass) over time are shown for each *Saccharomyces cerevisiae* strain. Scale bar indicates 50 µm. Data are means of *n* ≥ 3 independent experiments

Since Δ*sfl1* had significantly increased adhesion maintenance compared to WT cells, and rapamycin increased AMS through *EFG1* and *BCR1*, we searched the Candida Genome Database for cell-surface proteins regulated by these transcription factors. We identified *YWP1* (Yeast Wall Protein 1), a gene positively regulated by Efg1 and Sfl1^14^ (thus downregulated in our screening of these mutants). Ywp1 is a secreted yeast wall protein that plays a role in biofilm dispersion (presumably by associating with and disrupting other surface adhesion maintenance proteins). In this regard, Ywp1p-deficient yeast exhibited increased adhesiveness and biofilm formation on polystyrene plates.^19^ Thus, we expected that if Ywp1 interferes with other adhesion maintenance proteins, then *C. albicans* Δ*ywp1* cells would have improved biomass growth rates and increased AMS compared to WT cells in this flow system. Indeed, *C. albicans* Δ*ywp1* cells did initially show this phenotype of increased biomass accumulation and reduced cell detachment over the first 10 h of growth (Fig. 5, Supplementary Movie 10 and Table 2), as well as having significantly increased early AMS (Table 3). However, this phenotype was reversed in later biofilm growth so that *C. albicans* Δ*ywp1* cells had significantly reduced late AMS accompanied by increased late CDR (Table 3) resulting in decreased biofilm biomass (Fig. 5b). This biphasic effect suggested that Ywp1 does contribute to detachment in early biofilm growth (as previously shown for static biofilm growth), but may itself contribute to adhesion maintenance in later biofilm growth. This result also suggested that Ywp1 interacts with other *C. albicans* adhesin proteins expressed in early but not late biofilm growth.

Since the majority of *C. albicans* adhesins are temporally expressed and function cooperatively, we felt that single gene complementation would not clearly elucidate function. Instead, we took the approach of heterologous expression of *C. albicans* Ywp1 in a background of *S. cerevisiae* that does not express similar *C. albicans* adhesins. The closest homolog of *C. albicans YWP1* in *S. cerevisiae* (Flo5) has only 36% identity with 26% query cover, therefore it is unlikely *S. cerevisiae* expresses a functionally similar Ywp1 protein. Interestingly, *S. cerevisiae* WT cells (ScWT) had comparable early attachment to *C. albicans* WT cells (7.97 vs. 6.63% coverage area at 2 h respectively, *p* = 0.16 [coverage area was used for comparison between species as different imaging parameters did not allow direct comparison of cumulative adhesion]). Since the best characterized amyloid containing cell wall adhesin in *S. cerevisiae* is Flo11,^20–23^ we measured initial 2 h coverage area for a strain of *S. cerevisiae* with deletion of *FLO11*. This deletion strain showed nearly complete loss of initial attachment, such that by 2 h the *S. cerevisiae* Δ*flo11* strain had only 0.32% coverage area. Thus, Flo11 plays a major role in early attachment of ScWT cells in this flow system. However, over 18 h of the subsequent growth phase of biofilm formation, ScWT coverage area was significantly reduced compared to *C. albicans* WT cells (8.53% vs. 47.51%, *p* = 0.03), thus showing that Flo11 is not involved in adhesion maintenance and that ScWT cells lack most adhesion maintenance proteins expressed by *C. albicans* needed to develop a robust biofilm. Therefore, ScWT cells provided an ideal background to test expression of heterologous adhesin proteins such as *Sc-Ca*Ywp1. Immunoblot analysis of HA-epitope tagged *Sc-Ca*Ywp1 expressed from the *TEF2* promoter on a high-copy plasmid (PC7307) in wild-type *S. cerevisiae* (PC538) showed high heterologous expression of *Sc-Ca*Ywp1 (Supplementary Fig. 1).

Overall biofilm growth rates in *S. cerevisiae* cells differed from those of WT *C. albicans* cells in that two distinct rates were identified: the early growth phase that occurred from 0 to 10 h, and late growth phase from 10 to 18 h (Fig. 5c). Therefore, we compared ScWT cells with the *Sc-CaYWP1* strain for initial attachment, as well as AMS, as derived from CDR for early and late growth phases. The *Sc-CaYWP1* strain had a higher initial attachment rate than for ScWT cells (Fig. 5c, Table 4), suggesting that the presence of Ywp1 increases initial attachment, corroborating our results with *C. albicans*. Furthermore, the AMS of the *Sc-CaYWP1* strain was significantly higher (with CDR being significantly decreased) (Table 4) during the late growth phase but not the early growth phase compared to ScWT cells. We found the same results in each of the two independently constructed strains of *Sc-CaYWP1*. These data show that *C. albicans* Ywp1 has dual functions for both initial attachment and as an adhesion maintenance protein.

**Table 4:** Attachment and detachment of *Saccharomyces cerevisiae* under flow

	Attachment Rate^a^ (×10^5^)	Early CDR (0–10 h)^b^	Late CDR (10–18 h)^b^	Early AMS (0–10 h)^c^	Late AMS (10–18 h)^c^
ScWT	5.49 (0.12)	0.25 (0.01)	0.47 (0.04)	3.93	3.62
*Sc-CaYWP1*	8.89 (0.11)	0.28 (<0.01)^NS^	0.21 (0.01)	2.14	4.72

Each value represents a mean or best-fit slope of *n* ≥ 3 experiments, with standard deviation in parentheses

NS indicates no significant difference between each strain compared to wild-type cells. All other values were significant (*p* < 0.05)

^a^Attachment Rate represents the average biomass of newly attached cells /mm^2^/h within the first 2 h (the attachment phase). Cell detachment does not impact the attachment rate

^b^Cell Detachment Rate (CDR) was calculated as the average total detachment rate (average biomass of newly detached cells/mm^2^/h) divided by the biomass of biofilm for the first two hours. Values indicate the average proportion of biomass that detaches from the biofilm per hour

^c^Adhesion Maintenance Strength (AMS) was calculated as the inverse of the BDR. Value is reflective of the strength with which a cell remains adhered to the cells or substrate of a biofilm over time

## Discussion

*C. albicans* biofilms are initiated by adherence to host tissues while being subjected to shear forces generated by flowing fluids such as saliva, gastric juice, and blood. While many *C. albicans* adhesin proteins and their regulators have been studied, the mechanisms by which these cells adhere to a surface under flow are not known. Here, we gain a more detailed understanding of *C. albicans* biofilm development by temporal quantification of cellular adhesion events with flow.^4^ Although biofilm development in our flow system was carried out at 23 °C rather than 37 °C for more accurate quantitation, we previously found that flow itself induces over four-fold more gene expression changes compared to genes induced at 37 °C, including more changes of genes related to both adhesion and filamentation.^24^ While this limitation in our assay may make it difficult to extrapolate and compare to biofilms formed in other conditions, this flow model provides valuable information into adhesion and adhesins that regulate biofilm formation in *C. albicans*.

In contrast to most previous models of biofilm development in which cell dispersal occurs mainly after biofilm maturation, we found that dispersion in *C. albicans* biofilms is a continuous process beginning early in biofilm development. Subsequent to initial attachment, fungal cells detached from the substrate and maturing biofilm throughout the attachment and growth phases. Although fungal dispersion that occurs only in mature biofilms might happen in regions where *C. albicans* cells are completely protected from flow or shear forces, we and others^25^ found cell dispersion from biofilms with flow begins early and occurs constantly throughout biofilm development. Furthermore, dispersed cells have differences in their transcriptome associated with enhanced virulence characteristics and drug resistance,^26^ as well as higher expression of transporters needed for nutrient acquisition.^26^ Our observations concur with those of Uppuluri group that dispersed cells arise primarily from lateral yeast budding from hyphae rather than from dense cell groups typically released in response to quorum signaling between cells.^25^ Indeed, we noted that biofilms in late phase growth with high cell densities had similar rates of detachment per unit biomass as for smaller biofilms with more separated cells, indicating that dispersion is largely independent from close cell-cell communication.

Our study also revealed the importance of both initial adhesions and adhesion maintenance for development of *C. albicans* biofilm biomass. Unexpectedly, we found that deletion of single proteins in *C. albicans* (illustrated by strains Δ*hwp2*, Δ*ihd1*, and Δ*eap1*) resulted in nearly complete loss of initial attachment and collapse of biofilm development, while deletion of *C. albicans* adhesins Als1 and Als3, and Hyr1 resulted in less severe reduction of attachment and biofilm biomass (Fig. 2b and Table 1). In examining common features among these tested adhesin proteins, we observed that most contain discrete amyloidogenic regions either previously described (Als1, Als3, Eap1, Hwp2)^20,27^ or are computationally predicted (Hyr1, Supplementary Data 1). We suspect that during adhesion, the amyloid regions among the various adhesins may be interacting with each other, which would explain the observed inter-dependency of the adhesins.

In order to form a dense mature biofilm, *C. albicans* must remain adhered to a substratum over extended time periods. Our results show that different fungal proteins are responsible for initial attachment and for adhesion maintenance, particularly long-term adhesion maintenance. This is best illustrated by *C. albicans* Δ*efg1* and Δ*bcr1* cells, which despite having near WT levels of initial attachment, did not remain adhered over time (Fig. 4b and Table 1). Similarly, Δ*hog1* and Δ*sfl1* cells had significantly higher late phase AMS, despite having lower initial attachment rates. Thus, we expect that following initial attachment, various environmental stimuli that induce hyphal formation also alter expression of various cell wall proteins, including Ywp1, to change the AMS of the cell. Our finding that cells lacking *YWP1* (*C. albicans* Δ*ywp1* and WT *S. cerevisiae* that does not express a*YWP1* homolog) had lower AMS during late phase growth than their *YWP1* expressing counterparts (WT *C. albicans* and *Sc-CaYWP1*, respectively, Table 1 and Table 4), while only having minimal effect on initial attachment, indicates that this protein plays a unique role in altering the AMS of the cell. We conjecture that Ywp1 interferes with other, likely stronger, *C. albicans* adhesin proteins during early growth since Δ*ywp1* cells showed an increase in short-term adhesion maintenance during early biofilm formation (Table 3). This is specific to *C. albicans* adhesins as *Sc-CaYWP1* cells showed no change in short-term adhesion maintenance compared to WT *S. cerevisiae* cells (Table 4). It is possible that in *C. albicans* Ywp1 masks binding sites of other adhesins, preventing their attachment. It could also be interacting directly with other adhesins and masking their binding domains. This increased early adhesion maintenance corroborates previous studies of Δ*ywp1* cells that showed increased adhesion of this strain to polystyrene substratum.^19,28^

Two characteristics of Ywp1 may be involved in the mechanism by which it alters the AMS of the cell. First, Ywp1 is known to have a pro-peptide region of approximately 100 amino acids that is cleaved from the N-terminus and then re-associates with the protein. Second, it has been shown that Ywp1 contains five amyloidogenic regions, two of which are located on the pro-peptide region, that currently have no known role in the protein’s function.^28^ It is possible that the pro-peptide, the amyloid sequences, or both are involved in stabilizing adhesion to a substrate over extended periods.

**Fig. 6:**
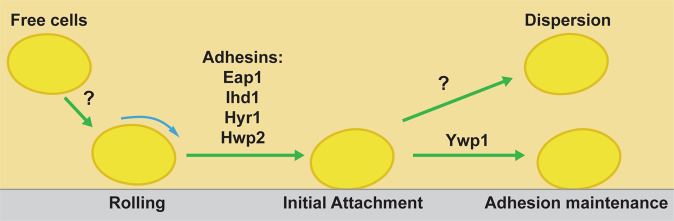
A model of the adhesion process of *Candida albicans* under flow. A multi-phase model of yeast cell adhesion under flow is shown. Free *C. albicans* cells in solution initially form weak interactions to the substrate surface through an unknown mechanism, resulting in cell rolling. This is followed by an initial attachment to the substrate surface, mediated through the action of numerous adhesin proteins, resulting in the halt of cell movement. At this time, environmental sensing is involved in determining the decision of the cell to either detach from the substrate and disperse, or commit to the present location and maintain adhesion. Long-term maintenance of adhesion is at least partly mediated through the action of Ywp1, while dispersion is mediated through an unknown mechanism

Together, our results suggest that the adhesion of *C. albicans* to a substratum is a multi-phase process (Fig. 6). This process begins with rolling of *C. albicans* across the surface, which may expose amyloid regions of initial attachment proteins and promote adhesion. Following initial adhesion, numerous environmental cell signaling events coordinate to determine whether long-term adhesion will be maintained, or if cells will disperse. Filamentation is important for the expression of adhesion maintenance proteins as demonstrated by the poor adherence of hyphal defective strains Δ*efg1* and Δ*bcr1* (Fig. 4), as well as the robust biofilm formation of the hyperfilamentous strains Δ*hog1* and Δ*sfl1* (Fig. 3). While we did not directly test the role of matrix formation in cell adhesion, our finding that rapamycin treatment of cells significantly increased AMS points to a role for the matrix since rapamycin is known to increase expression of several essential matrix formation genes by 4–7 fold unrelated to temperature or media.^15^ Long-term adhesion maintenance to a substratum is at least partially mediated by Ywp1 (Fig. 5), making this protein an adhesion maintenance cell wall protein of *C. albicans*.

## Methods

### Strains and growth media

Yeast strains used in this study are summarized in Table S1. Cultures were grown overnight in 1% (w/v) yeast extract, 2% (w/v) bacto peptone, and 2% (w/v) glucose (YPD, Difco, Detroit, MI) supplemented with 50 mg/ml of uridine (Sigma-Aldrich). Cell number from overnight cultures was determined using a haemocytometer, and values were used to determine volumes of overnight culture to add to the attachment flask to reach 1 × 10^6^ cells/ml. After inoculation, cells were allowed to acclimate for 15 min prior to initiation of flow. For *S. cerevisiae* growth and transformants selection, Synthetic Complete (SC) media consisted of 0.67% Yeast Nitrogen Base (YNB) with ammonium sulfate (MP Biomedicals®), 2% glucose, and 0.08% of Complete Supplement Mixture without Uracil (MP Biomedicals®) was used.

### Heterologous expression of *C. albicans YWP1* in *S. cerevisiae*

For the analysis of *C. albicans* Ywp1 in *S. cerevisiae*, we chose *S. cerevisiae* PC538 strain^29^ as a surrogate host. The *Sc-CaYWP1* gene was expressed under the control of p*TEF2* promoter using the plasmid vector YEp352.^30^ YEp352 containing the p*TEF2* promoter has been described.^31^ The amino acid sequence of *C. albicans YWP1* (orf19.3618) was obtained from the Candida Genome Database (www.candidagenome.org) and translated to nucleic acid sequence using the *S. cerevisiae* codon usage table on the gene platform Invitrogen™ GeneArt™ Strings™ DNA Fragments Service (Thermo Fisher Scientific, San Jose, CA, USA). Nucleotide sequences flanking the codon-optimized *Sc-CaYWP1* contained a 5’ XbaI site and a 3’ SalI site to facilitate cloning into the YEp352-p*TEF2* vector. Two restriction sites were also designed in the *YWP1* gene at positions 249 bp (from the translational start site) (SacII) and position 423 bp (XhoI) to allow for subsequent insertion of an epitope at these positions. The internal restriction sites led to two changes: one at amino acid position 84 (from alanine to arginine) and a second at amino acid position 143 (from isoleucine to serine), respectively. The sequence of entire *Sc-CaYWP1* gene was custom-synthesized by using the Invitrogen™ GeneArt™ Strings™ DNA Fragment Service (Thermo Fisher Scientific, San Jose, CA, USA, Supplementary Data 2).

The custom-made *Sc-CaYWP1* fragment was digested with XbaI and SalI and cloned into XbaI and SalI digested YEp352-p*TEF2* vector (PC6365) to create YEp352-p*TEF2*-*Sc-CaYWP1* (*PC7155*). Cloning was confirmed with DNA sequencing analysis at Genewiz sequencing facility (South Plainfield, NJ). The plasmid was introduced into *S. cerevisiae* strains using standard transformation procedures.^32,33^ The plasmid was introduced into two strains, wild type (PC538) and the *ScΔflo11* mutant (PC1029). Transformants were selected into synthetic drop out media lacking uracil, SD-URA (0.67% YNB, 2% Dextrose, 1X amino acids). Two independent transformants from each strain background were used in subsequent flow experiments.

The plasmid containing an HA-epitope-tagged version of *Sc-CaYWP1* was also constructed, called YEp352-p*TEF2*-*Sc-CaYWP1*-HA (PC7303). The plasmid was constructed from YEp352-p*TEF2*-*Sc-CaYWP1* (*PC7155*) by a homologous recombination approach in *S. cerevisiae*. In particular, a DNA fragment was designed that contained the gene encoding the hemagglutinin (HA) epitope with flanking sequences to construct an in-frame fusion with *Sc-CaYWP1* at position 423 bp, at the site where XhoI site was introduced. Complementary primers were designed 5'-GAAACTCCAATCGTTAAGAGAGATCAAATCGACGATTTCATTGCCTACCCATACGATGTTCCTGACTATGCGGGCTATCCGTATGACGTCCCGGACTATGCAGGATCCTATCCATATGACGTTCCAGATTACGCTTCGAGTGAAAACACTGAAGGTACTGCTTTGGAAGGTTCTACATTG-3' and 5'-CAATGTAGAACCTTCCAAAGCAGTACCTTCAGTGTTTTCACTCGAAGCGTAATCTGGAACGTCATATGGATAGGATCCTGCATAGTCCGGGACGTCATACGGATAGCCCGCATAGTCAGGAACATCGTATGGGTAGGCAATGAAATCGTCGATTTGATCTCTCTTAACGATTGGAGTTTC-3' (Millipore Sigma, St. Louis, MO) that contained the gene encoding the HA sequence flanked by the *Sc-CaYWP1* sequence, to target integration into the XhoI digested YEp352-p*TEF2*-*Sc-CaYWP1* plasmid. Approximately 6 μg of each primer was mixed in a 25 μl volume and hybridized by 2 cycles of heating (at 98 °C for 10 min) and cooling (at 24 °C for 10 min). Hybridization of the two long primers was confirmed by agarose gel electrophoresis before storing the double stranded hybridized DNA fragment at −20 °C.

To construct YEp352-p*TEF2*-*Sc-CaYWP1*-HA, the YEp352-p*TEF2*-*Sc-CaYWP1* plasmid was linearized by digestion with XhoI and co-transformed with hybridized DNA fragment encoding the HA epitope into PC538 (auxotrophic for uracil). Transformants were selected onto SD-URA media. After initial screening by immunoblot analysis, plasmids were rescued from four independent transformants. Cells were grown to saturation in 10 ml SD-URA media. Promega Wizard Plus SV Miniprep DNA Purification system (A1460) was used with the following modification. Glass beads (100 μl) were added at lysis step, and cells were vortexed for 5 min before the addition of neutralization buffer. Rescued plasmids were transformed into *E. coli*. Positives clones were confirmed by DNA sequencing and immunoblot analysis.

### Immunoblot analysis

Immunoblot analysis was performed to validate expression levels of Sc-CaYwp1. All blots were derived from the same experiment and processed in parallel. Cells were grown to mid-log in 10 ml SD-URA, and pellets were washed once with water. Pellets were re-suspended in 200 μl Thorner Buffer (40 mM Tris pH 8, 5% SDS, 8 M Urea, 100 μM EDTA, 0.4 mg/ml bromophenol blue) and vortexed with 100 μl glass beads for 5 min. Cell extracts were boiled for 5 min at 98 °C and centrifuged at 15,900×*g* for 5 min. Supernatants were loaded onto sodium dodecyl sulfate (SDS) polyacrylamide gel electrophoresis (SDS-PAGE) gels (12% acrylamide) and transferred to nitrocellulose membranes. Membranes were blocked with 5% non-fat dried milk and probed with α-HA primary antibodies (1:5000 dilution in blocking buffer, clone 12CA5, Roche, Basel, Switzerland, 11583816001). Mouse α-Pgk1 (1:5000 dilution in blocking buffer, Novex, Winston-Salem, NC, USA, 459250) antibodies were used as a control for total protein levels. Goat α-mouse secondary antibodies were used to detect primary antibodies (1:5000 dilution in blocking buffer, Bio-Rad, Hercules, CA, USA, 170-6516). Blots were visualized by chemiluminescence using a Bio-Rad ChemiDoc XRS + system (Bio-Rad, 1708265).

### Flow System

Flow experiments were split into two phases by using two separate media flasks. During the first phase (attachment phase), fresh YPD seeded with *C. albicans* cells (1 × 10^6^ cells/ml) was circulated through a µ-Slide I 0.8 Luer family ibiTreat flow chamber (ibidi, Martinsried, Germany) using a peristaltic pump. This phase proceeded for 2 h, during which time cells were able to attach to the coverslip surface of the flow chamber. Afterwards, the source of media to the slide was switched to cell-free YPD for the remainder of the experiment (growth phase). The return flow during the growth phase was passed through four sequential cell filters, first two coarse filters (20 µm and 10 µm pore size, Analytical Scientific Instruments, Richmond, CA), then a 2 µm pore HPLC filter (Sigma Aldrich, St. Louis, MO) followed by a 0.22 µm PVDF filter (Sterivex™, Millipore, Billerica, MA, USA), before being recycled so as to prevent contamination of the stock medium. Thus, during the attachment phase, cells are allowed to re-circulate across the surface of the slide, but during the growth phase all cells are removed prior to re-circulation, and media to the slide remains cell free for the rest of the experiment.

In all experiments, flow was set to generate a shear force of 0.8 dynes/cm^2^ across the surface of the flow chamber. This value has been previously calculated as the approximate shear force that human saliva exerts in the oral cavity.^34^ A hotplate stirrer with an external temperature probe was used to warm the attachment flask to 37 °C. The microscope, including the slide being imaged and several feet of preceding tubing, were maintained at 23 °C for the duration of the experiment.

For *S. cerevisiae* flow experiments, biofilm growth conditions were optimized to facilitate optimal growth of *S. cerevisiae* strains. The experiments were carried out in SC media without uracil and at a temperature of 30 °C throughout the course of the experiment.

### Imaging

All images were taken using a Zeiss AxioScope A.1 transmitted light microscope (Zeiss, Göttingen, Germany) with dark field illumination. Imaging conditions were maintained between experiments to allow for quantitative comparisons. Due to differences in cell size and apparent cell brightness, imaging conditions were adjusted for *S. cerevisiae* experiments, therefore quantitative comparisons cannot be made directly between *C. albicans* data and *S. cerevisiae* data. For all experiments, images were acquired every 2 min during the attachment phase and every 15 min during the growth phase.

### Image analyses

Image analysis was conducted in the ImageJ software environment^35^ after conversion to an 8-bit grayscale format. Statistical analyses, including linear regressions, were performed in Graphpad Prism® version 5.03 software.

To determine the coverage area of the biofilm, thresholds were applied to every image at a gray value minimum of 15, and percent surface area was measured. To evaluate the biomass of the attached cells (biofilm biomass) a densitometry analysis was performed. Specifically, the cumulative gray values of all pixels above 15 were evaluated for every frame of the dark field time-lapse movies, and then normalized to the imaging area. Growth rate of each biofilm was evaluated by fitting an unconstrained linear regression to all biomass data collected.

To evaluate the rate of cell attachment during the attachment phase, a given frame (*n*) was subtracted from its next frame (*n* + 1) for every image of the attachment phase [(*n* + 1)−*n*]. This subtraction resulted in a time-lapse of attachment where any cell that attached to the imaging region between each frames remain bright (at the intensity contributed by that cell), while cells that remained constant or detached between frames were removed. This resulted in an attachment stack, a time-lapse movie of every attaching cell as it attached to the biofilm or substrate. A threshold was then applied to these calculated images, highlighting newly attached cells, and subsequently processed using the ImageJ binary erosion filter to limit background noise and minor shifts in cell position. In addition, to increase specificity towards cells, detected particles had to be a minimum of 20 µm^2^ (4.5 µm^2^ for *S. cerevisiae*). This generated mask was then applied to the attachment stack, and the biomass of the attached cells at every frame was measured as described above. Rates of cell attachment were then determined by fitting the cumulative biomass of attaching cells for the first 2 h with unconstrained linear regressions.

The rate of detachment was determined in a similar manner to attachment, but the image subtraction was reversed [*n*−(*n* + 1)], resulting in an image that highlighted cells that had detached between frames. Detachment rates were evaluated over the duration of the experiment (attachment and growth phases) in a manner similar to attachment rates. The rate of total cell detachment was found to increase with increasing biomass of the biofilm, which may arise due to the increased number of cells available to detach. Thus, values obtained for this variable did not reflect the relative ease with which cells were removed from the biofilm or substrate surface, which was an important parameter to consider. Thus, we normalized the biomass of detaching cells obtained between each frame to the total biomass of the biofilm prior to these detachments ([*n*−(*n* + 1)]_biomass_/*n*_biomass_), resulting in a value that represents the proportion of cells that detached (referred to as cell detachment). The rates of cell detachment were also evaluated using unconstrained linear regressions on the cumulative value over time. The adhesion maintenance strength was derived by taking the inverse of the regression value of the cell detachment.

To estimate relative cell-cell to cell-surface binding strengths, we performed image subtraction [(*n* + 1)−*n*] to determine newly attached cells at each frame, and applied a threshold to these images (as described above). These images were then processed using the ImageJ binary erosion filter, and particles at least 20 µm^2^ and with a circularity value of at least 0.4 were counted as cells. These particles were then compared to images of the biofilm coverage area of the preceding frame (*n*), to determine regions of overlap (completed using the ‘AND’ operator in the ImageJ image calculator). Regions of overlap were counted as cell–cell adhesion events if they were at least 2.5 µm^2^. The number of cell–cell adhesions was then normalized to the total number of adhesion events per frame, giving the relative cell–cell adhesion. If there were no adhesion events in a frame, no data was entered for that time point.

To better illustrate the relative impacts of the attachment rates and biomass detachment rates, we empirically determined a predictive formula for the biomass at 2 h:

1 $$B = 2.1A \times (1.36-D)$$

Where, *B* is the predicted biomass at 2 h, *A* is the attachment rate, and *D* is the cell detachment rate. The 2.1 constant value largely reflects the 2 h of attachment time, so that the growth rate is effectively incorporated into the A value in the equation. This equation illustrates the importance of the cell detachment rate for development of total biomass during the Attachment phase. Cell detachment values above 0.36 will result in a decrease in overall biomass, while values below 0.36 will result in an increase in 2 h biomass. While this equation is specific to the parameters of this flow system (time, flow rate and growth conditions), it shows that the detachment rate is the critical factor for development of the biomass.

### Statistical comparisons

All the graphs for attachment, detachment and biomass detachment were illustrated using cumulative values due to the extensive frame to frame variation for these values. Graphs of the non-cumulative values at each frame were erratic and had extensive overlaps between strains, making them difficult to compare. All regression analyses were unconstrained (not forced through the origin), and non-overlapping 95% confidence intervals were considered statistically significant at *p* < 0.05.

### Reporting summary

Further information on research design is available in the Nature Research Reporting Summary.

## Supplementary information

Reporting Summary Checklist

Supplementary Material

Supplemental Movie 1

Supplemental Movie 2

Supplemental Movie 3

Supplemental Movie 4

Supplemental Movie 5

Supplemental Movie 6

Supplemental Movie 7

Supplemental Movie 8

Supplemental Movie 9

Supplemental Movie 10

## Data Availability

The data that support the findings of this study are available from the corresponding author upon reasonable request.

## Supplementary information

**Supplementary information** accompanies the paper on the *npj Biofilms and Microbiomes* website (10.1038/s41522-019-0094-5).

## Code availability

The ImageJ scripts utilized for image analyses are available from the corresponding author upon reasonable request.
