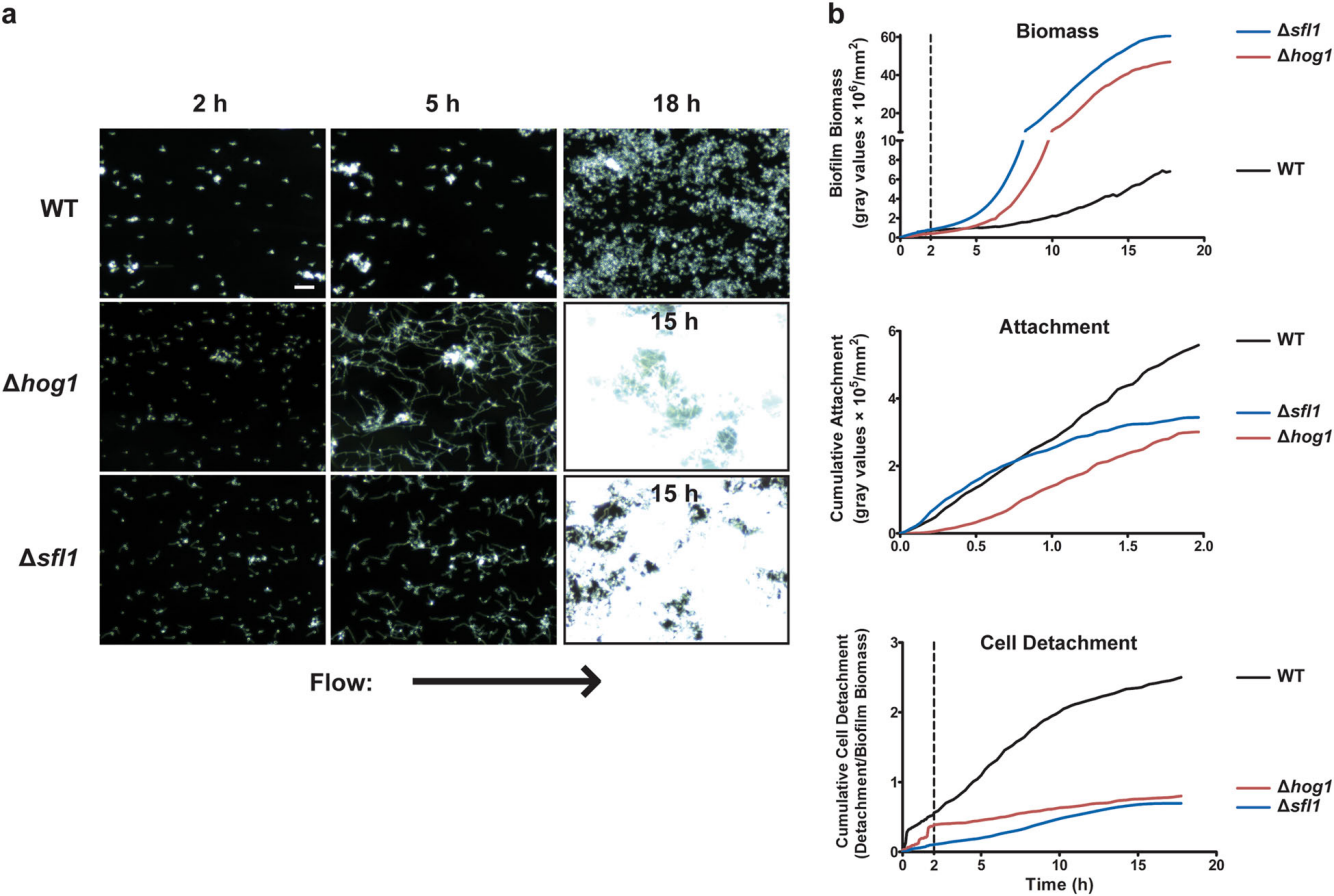# Author Correction: *Candida albicans* biofilm development is governed by cooperative attachment and adhesion maintenance proteins

**DOI:** 10.1038/s41522-021-00264-x

**Published:** 2021-12-16

**Authors:** Andrew D. McCall, Ruvini U. Pathirana, Aditi Prabhakar, Paul J. Cullen, Mira Edgerton

**Affiliations:** 1 Department of Oral Biology, School of Dental Medicine, The State University of New York at Buffalo, Buffalo, NY 14214 USA; 2 Department of Biological Sciences, College of Arts and Sciences, The State University of New York at Buffalo, Buffalo, NY 14214 USA

**Keywords:** Biofilms, Pathogens

Correction to: *npj Biofilms and Microbiomes* 10.1038/s41522-019-0094-5, published online 23 August 2019

While selecting representative images for publication from the same time-lapse experiment, the authors mistakenly selected images they had published previously^1^ in Figs 1a and 3 (hog1 panels). These images have now been replaced with equivalent images from the same experiment, but that have not been published previously.

**Figure 1:**
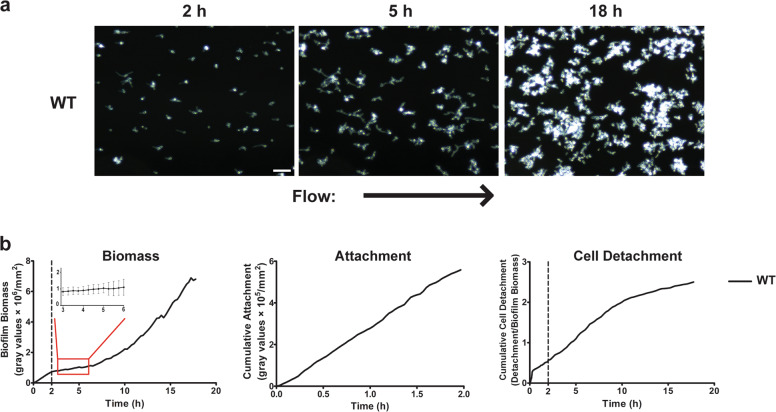

**Figure 2:**