# Supplementary material for: Candida albicans biofilm development is governed by cooperative attachment and adhesion maintenance proteins
Source: NPJ Biofilms Microbiomes. 2019 Aug 23;5:21. doi: 10.1038/s41522-019-0094-5 (PMC6707306; doi:10.1038/s41522-019-0094-5)
Supplement: Supplementary file 2 — Supplementary Material [file 41522_2019_94_MOESM2_ESM.pdf]

Supplementary Data 1. Amyloid regions of Hyr1

1 MKVVSN**FIFT** **ILL**TLNLSAA LEVVTSRIDR GGIQGFHGDV KVHSGATWAI

51 LGTTLCSFFG GLEVEKGASL FIKSDNGPVL ALNVALSTLV RPVINNGVIS

101 LNSKSSTSFS NFDIGGSSFT NNGEIYLASS GLVKSTAYLY AREWTNNGLI

151 VAYQNQKAAG NIAFGTAYQT ITNNGQICLR HQDFVPATKI KGTGCVTADE

201 DTWIKLGNTI LSVEPTHNFY LKDSKSSLIV HAVSSNQTFT VHGFGNGNKL

251 GLTLPLTGNR DHFRFEYYPD TGILQLRAAA LPQYFKIGKG YDSKLFRIVN

301 SRGLKNAVTY DGPVPNNEIP AVCLIPCTNG PSAPESESDL NTPTTSSIET

351 SSYSSAATES SVVSESSSAV DSLTSSSLSS KSESSDVVSS TTNIESSSTA

401 IETTMNSESS TDAGSSSISQ SESSSTAITS SSETSSSESM SASSTTASNT

451 SIETDSGIVS QSESSSNALS STEQSITSSP GQSTIYVNST VTSTITSCDE

501 NKCTEDVVTI FTTVPCSTDC VPTTGDIPMS TSYTQRTVTS TITNCDEVSC

551 SQDVVTYTTN VPHTTVDATT TTTTSTGGDN STGGNESGSN HGSGAGSNEG

601 SQSGPNNGSG SGSEGGSNNG SGSGSDSGSN NGSGSGSNNG SGSGSNNGSG

651 SGSGSTEGSE GGSGSNEGSN HGSNEGSGSG SGSQTGSGSG SNNGSGSGSQ

701 SGSGSGSQSG SESGSNSGSN EGSNPGAGNG SNEGSGQGSG NGSEAGSGQG

751 SGPNNGSGSG HNDGSGSGSN QGSNPGAGSG SGSESGSNAG SHSGSNEGAK

801 TDSIEGFHTE SKPGFNTGAH TDATVTGNSV ANPVTTSTES DTTISVTVSI

851 TSYMTGFDGK PKPFTTVDVI PVPHSMPSNT TDSSSSVPTI DTNENGSSIV

901 TGGKS**ILFGL** **IVSMVVL**FM

Supplementary Data 1. The amino acid sequence of Hyr1 with regions predicted to be amyloidogenic regions by Waltz webserver were underlined (http://waltz.switchlab.org), and predictions by TANGO were bolded (http://tango.crg.es). For both analyses, default parameters were used, and only β-aggregation potentials of at least 97% are illustrated.

Supplementary Data 2. Synthesized *Sc-CaYWP1* Sequence:

AGCGATCATGTCTAGATGAAGGTTTCCACTATTTTTGCTGCTGCTTCTGCTTTGTTTGCTGCTAC
TACTACTTTGGCTCAAGATGTTGCTTGCTTGGTTGATAATCAACAAGTTGCCGTTGTTGATTTGG
ATACTGGTGTTTGCCCTTTTACTATTCCAGCTTCTTTGGCTGCTTTCTTCACTTTCGTTTCTTTG
GAAGAGTACAACGTCCAATTCTACTACACCATCGTTAACAACGTTAGATACAACACCGATATTAG
AAACCGCGGTAAGGTTATTAACGTTCCAGCTAGAAACTTGTATGGTGCTGGTGCTGTTCCATTTT
TCCAAGTTCATTTGGAAAAGCAGTTGGAAGCTAATTCTACTGCTGCTATTAGACGTAGATTGATG
GGTGAAACTCCAATCGTTAAGAGAGATCAAATCGACGATTTTATTGCCTCGAGTGAAAACACTGA
AGGTACTGCTTTGGAAGGTTCTACATTGGAAGTTGTTGACTATGTTCCAGGTTCTTCTTCTGCTT
CTCCATCTGGTTCAGCTTCACCATCAGGTTCTGAATCTGGTTCTGGTAGTGATTCTGCTACCATT
AGATCTACTACCGTTGTCTCTTCTTCCTCTTGTGAATCTTCTGGTGATTCAGCTGCTACAGCTAC
TGGTGCTAATGGTGAATCTACTGTTACTGAACAAAACACCGTTGTTGTTACCATTACCTCTTGTC
ATAACGATGCTTGTCATGCTACTACAGTTCCAGCAACTGCTTCTATTGGTGTTACTACTGTTCAT
GGTACTGAAACCATTTTCACTACCTACTGTCCATTGTCCTCTTACGAAACTGTTGAATCCACCAA
AGTTATCACTATCACCTCTTGCTCTGAAAACAAGTGTCAAGAAACTACTGTTGAAGCTACTCCAT
CTACTGCTACAACTGTTTCTGAAGGTGTTGTCACTGAATACGTTACTTACTGTCCAGTTTCTTCC
GTTGAAACCGTTGCTTCTACAAAGGTCATTACTGTTGTTGCTTGTGATGAACATAAGTGCCACGA
AACTACAGCTGTTGCTACTCCAACTGAAGTTACAACAGTTGTTGAAGGTTCCACCACTCATTATG
TTACTTATAAGCCAACAGGTTCCGGTCCAACTCAAGGTGAAACTTATGCTACTAATGCCATCACA
AGTGAAGGTACAGTTTACGTTCCAAAGACTACTGCTGTTACTACACATGGTTCTACTTTTGAAAC
TGTCGCCTACATTACAGTTACTAAGGCTACACCAACAAAAGGTGGTGAACAACATCAACCAGGTT
CTCCAGCTGGTGCCGCTACTTCTGCTCCAGGTGCTCCTGCTCCTGGTGCATCTGGTGCTCATGCT
TCAACTGCTAACAAAGTTACCGTTGAAGCTCAAGCTACACCAGGTACTTTGACTCCAGAAAACAC
AGTTGCCGGTGGTGTTAACGGTGAACAAGTAGCTGTTTCTGCTAAGACTACTATTTCTCAAACTA
CCGTCGCTAAAGCTTCAGGTTCAGGTAAAGCTGCTATTTCTACTTTCGAAGGTGCTGCTGCAGCT
TCCGCTGGTGCTTCTGTTTTAGCTTTGGCTTTGATTCCATTGGCCTACTTCATTTGAGTCGACAT
CTTATACGACTA

Amplification Primers for above sequence:

Forward:
5'-AGCGATCATGTCTAGATGAAGGTTTCCACT-3'

Reverse:
5'-TAGTCGTATAAGATGTCGACTCAAATGAAGTAGGCCA-3'

Supplementary Data 2. The *Sc-CaYWP1* gene and amplification primers were custom-synthesized using Invitrogen™ GeneArt™ Strings™ DNA Fragment Service.

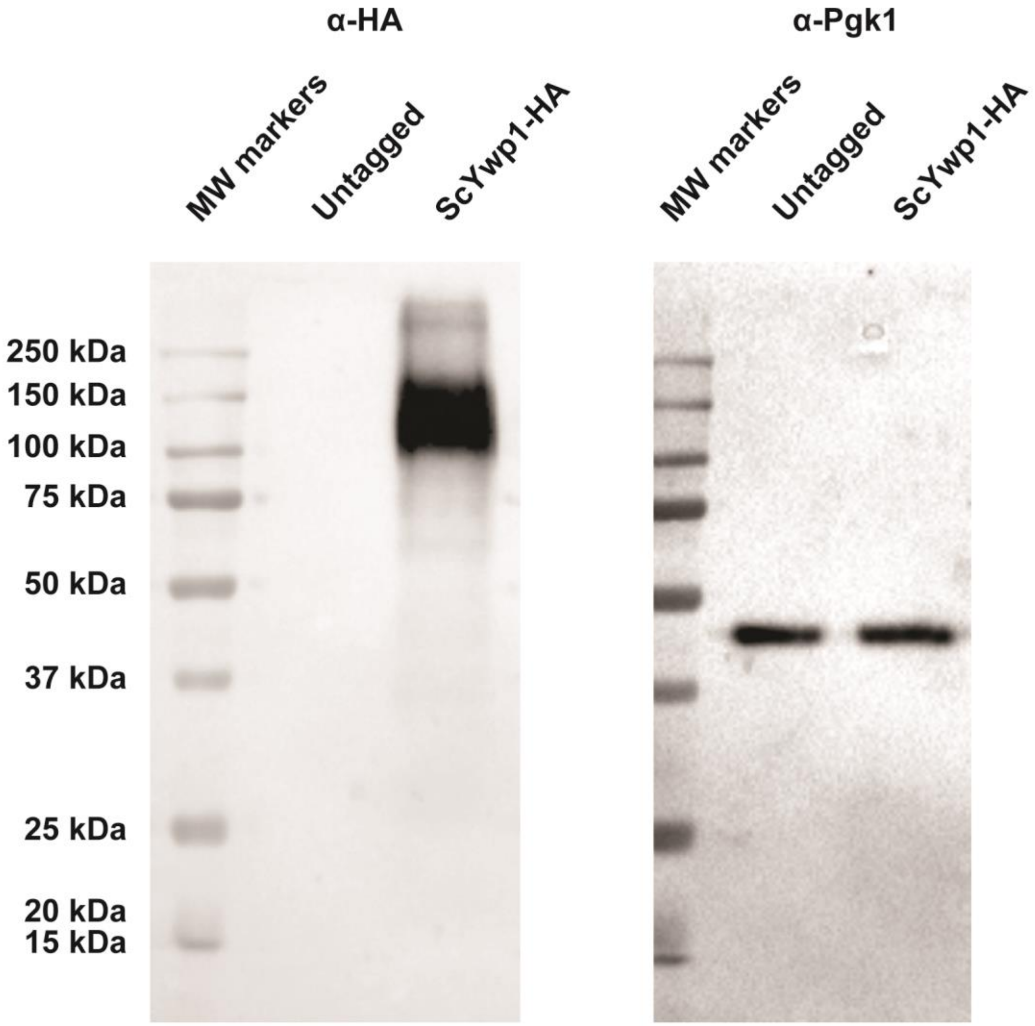

Supplementary Figure 1. Heterologous expression of *Sc-CaYWP1* in *S. cerevisiae*.

Immunoblot analysis of untagged (PC7155) and HA-epitope tagged *Sc*-*Ca*Ywp1 expressed from *TEF2* promoter on a high-copy plasmid (PC7307) in wild-type *S. cerevisiae* (PC538). The HA-*Sc-Ca*Ywp1 protein may run at a higher molecular weight (150 kDa) than its expected size (61 kDa) due to glycosyl modifications. Antibodies to Pgk1 (45 kDa) were used as a control for total protein levels.

Name	Strain	Genotype, Reference
Candida albicans WT	CAI4 + *URA*	*Δura3::imm434/Δura3::imm434, RPS1/Δrps1::Clp10-URA3*, [1]
SN250	SN250	*his1Δ/his1Δ, leu2Δ/leu2Δ, arg4Δ/arg4Δ, URA3/ura3Δ::imm434, IRO1/iro1Δ::imm434,* [2]
Δ/Δ*als1/3*	CJN1348	*ura3Δ::imm434:: URA3-IRO1 als1::hisG als3::dpl200 ura3Δ::imm434 als1::hisG als3::dpl200*, [3]
Δ*bcr1*	SN275	*his1Δ/his1Δ, leu2Δ/leu2Δ, arg4Δ/arg4Δ, URA3/ura3Δ::imm434, IRO1/iro1Δ::imm434, bcr1Δ::C.dubliniensisHIS1/bcr1Δ::C.maltosaLEU2*, [2]
Δ*efg1*	TF156-X	*his1Δ/his1Δ, leu2Δ/leu2Δ, arg4Δ/arg4Δ, URA3/ura3Δ::imm434, IRO1/iro1Δ::imm434, efg1Δ::C.dubliniensisHIS1/efg1Δ::C.maltosaLEU2*, [4]
Δ*hog1*	SN307	*his1Δ/his1Δ, leu2Δ/leu2Δ, arg4Δ/arg4Δ, URA3/ura3Δ::imm434, IRO1/iro1Δ::imm434, hog1Δ::C.dubliniensisHIS1/hog1Δ::C.maltosaLEU2*, [2]
Δ*hwp2*	SN73	*his1Δ/his1Δ, leu2Δ/leu2Δ, arg4Δ/arg4Δ, URA3/ura3Δ::imm434, IRO1/iro1Δ::imm434, hwp2Δ::C.dubliniensisHIS1/hwp2Δ::C.maltosaLEU2*, [2]
Δ*hyr1*	SN823	*his1Δ/his1Δ, leu2Δ/leu2Δ, arg4Δ/arg4Δ, URA3/ura3Δ::imm434, IRO1/iro1Δ::imm434, hyr1Δ::C.dubliniensisHIS1/hyr1Δ::C.maltosaLEU2*, [2]
Δ*ihd1*	SN917	*his1Δ/his1Δ, leu2Δ/leu2Δ, arg4Δ/arg4Δ, URA3/ura3Δ::imm434, IRO1/iro1Δ::imm434, ihd1Δ::C.dubliniensisHIS1/ihd1Δ::C.maltosaLEU2,* [2]
Δ*sfl1*	TF001-X	*his1Δ/his1Δ, leu2Δ/leu2Δ, arg4Δ/arg4Δ, URA3/ura3Δ::imm434, IRO1/iro1Δ::imm434, sfl1Δ::C.dubliniensisHIS1/sfl1Δ::C.maltosaLEU2*, [4]
Δ*ywp1*	SN630	*his1Δ/his1Δ, leu2Δ/leu2Δ, arg4Δ/arg4Δ, URA3/ura3Δ::imm434, IRO1/iro1Δ::imm434, ywp1Δ::C.dubliniensisHIS1/ywp1Δ::C.maltosaLEU2*, [2]
Saccharomyces cerevisiae WT	PC538	*MAT***a** *ste4 FUS1-lacZ FUS1-HIS3 ura3-52* Σ1278b, [5]
*Sc*Δ*flo11*	PC1029	*MAT***a** *ste4 FUS1-lacZ FUS1-HIS3 ura3-52*) *flo11*::KanMX6 Σ1278b, [6]
Sc-CaYWP1	PC7155	Σ1278b *MAT***a** *ste4 FUS1-lacZ FUS1-HIS3 ura3-52 -scYWP1*, This paper

Supplementary Table 1. Strains used in the study. All deletion strains for *Candida albicans* were homozygous knockouts. Δ/Δ*als*1/3 is a dual homozygous knockout strain of *ALS1* and *ALS3*.

References for Supplementary Table 1.

1. Fonzi, W. A. & Irwin, M. Y. Isogenic strain construction and gene mapping in *Candida albicans*. *Genetics* **134**, 717–28 (1993).

2. Noble, S. M., French, S., Kohn, L. A., Chen, V. & Johnson, A. D. Systematic screens of a *Candida albicans* homozygous deletion library decouple morphogenetic switching and pathogenicity. *Nat. Genet.* **42**, 590–8 (2010).

3. Nobile, C. J. *et al.* Complementary adhesin function in *C. albicans* biofilm formation. *Curr. Biol.* **18**, 1017–24 (2008).

4. Homann, O. R., Dea, J., Noble, S. M. & Johnson, A. D. A phenotypic profile of the *Candida albicans* regulatory network. *PLoS Genet.* **5**, e1000783 (2009).

5. Cullen, P. J. *et al.* A signaling mucin at the head of the Cdc42- and MAPK-dependent filamentous growth pathway in yeast. *Genes Dev.* **18**, 1695–708 (2004).

6. Karunanithi, S. *et al.* Shedding of the mucin-like flocculin Flo11p reveals a new aspect of fungal adhesion regulation. *Curr. Biol.* **20**, 1389–95 (2010).

Supplementary Movie Legends

Supplementary Movie 1. Adhesion of WT cells under flow at 23°C. This time-lapse darkfield microscopy movie shows the attachment of CAI4 + *URA* cells to the substrate during the attachment phase (time indicated in the upper left hand corner; images acquired every 2 m), followed by the subsequent growth and development during the growth phase (starts at 2h; images acquired every 15 m). Cell-seeded media ($1{\times}10^6$ cells/ml) was used during the attachment phase, while cell-free media was used during the growth phase. Flow is from the right to left. Scale bar indicates 50 µm.

Supplementary Movie 2. Δ/Δ*als1/3* cells have reduced cell-cell adhesion under flow. This time-lapse darkfield microscopy movie shows the attachment of Δ/Δ*als1/3* cells to the substrate during the attachment phase (time indicated in the upper left hand corner; images acquired every 2 m), followed by the growth phase (starts at 2h; images acquired every 15 m). Cell-seeded media ($1{\times}10^6$ cells/ml) was used during the attachment phase, while cell-free media was used during the growth phase. Flow is from the right to left. Scale bar indicates 50 µm.

Supplementary Movie 3. Δ*hyr1* cells have reduced adhesion under flow. This time-lapse darkfield microscopy movie shows the attachment of Δ*hyr1* cells to the substrate during the attachment phase (time indicated in the upper left hand corner; images acquired every 2 m), followed by the growth phase (starts at 2h; images acquired every 15 m). Cell-seeded media ($1{\times}10^6$ cells/ml) was used during the attachment phase, while cell-free media was used during the growth phase. Flow is from the right to left. Scale bar indicates 50 µm.

Supplementary Movie 4. Only a few Δ*hwp2* cells adhere under flow. This time-lapse darkfield microscopy movie shows the attachment of Δ*hwp2* cells to the substrate during the attachment phase (time indicated in the upper left hand corner; images acquired every 2 m), followed by the growth phase (starts at 2h; images acquired every 15 m). Cell-seeded media ($1{\times}10^6$ cells/ml) was used during the attachment phase, while cell-free media was used during the growth phase. This Movie is also reflective of results obtained for Δ*eap1* and Δ*ihd1* cells*.* Flow is from the right to left. Scale bar indicates 50 µm.

Supplementary Movie 5. *Δhog1* cells show robust hyperfilamentation and remain strongly adhered over time under flow. This time-lapse darkfield microscopy movie shows the attachment of Δ*hog1* cells to the substrate during the attachment phase (time indicated in the upper left hand corner; images acquired every 2 m), followed by the growth phase (starts at 2h; images acquired every 15 m). Cell-seeded media ($1{\times}10^6$ cells/ml) was used during the attachment phase, while cell-free media was used during the growth phase. Flow is from the right to left. Scale bar indicates 50 µm.

Supplementary Movie 6. *Δsfl1* cells show robust hyperfilamentation and remain strongly adhered over time under flow. This time-lapse darkfield microscopy movie shows the attachment of Δ*sfl1* cells to the substrate during the attachment phase (time indicated in the upper left hand corner; images acquired every 2 m), followed by the growth phase (starts at 2h; images acquired every 15 m). Cell-seeded media (1×10^6 cells/ml) was used during the attachment phase, while cell-free media was used during the growth phase. Flow is from the right to left. Scale bar indicates 50 µm.

Supplementary Movie 7. *Δefg1* cells fail to remain adhered over time under flow. This time-lapse darkfield microscopy movie shows the attachment of Δ*efg1* cells to the substrate during the attachment phase (time indicated in the upper left hand corner; images acquired every 2 m), followed by the growth phase (starts at 2h; images acquired every 15 m). Cell-seeded media (1×10^6 cells/ml) was used during the attachment phase, while cell-free media was used during the growth phase. Flow is from the right to left. Scale bar indicates 50 µm.

Supplementary Movie 8. *Δbcr1* cells fail to remain adhered over time under flow. This time-lapse darkfield microscopy movie shows the attachment of Δ*bcr1* cells to the substrate during the attachment phase (time indicated in the upper left hand corner; images acquired every 2 m), followed by the growth phase (starts at 2h; images acquired every 15 m). Cell-seeded media (1×10^6 cells/ml) was used during the attachment phase, while cell-free media was used during the growth phase. Flow is from the right to left. Scale bar indicates 50 µm.

Supplementary Movie 9. WT cells treated with rapamycin (20 nM) have increased adhesion maintenance over time. This time-lapse darkfield microscopy movie shows the attachment of WT cells to the substrate during the attachment phase (time indicated in the upper left hand corner; images acquired every 2 m), followed by the growth phase (starts at 2h; images acquired every 15 m). Cell-seeded media (1×10^6 cells/ml) with 20 nM rapamycin was used during the attachment phase, while cell-free media with 20 nM rapamycin was used during the growth phase. Flow is from the right to left. Scale bar indicates 50 µm.

Supplementary Movie 10. *Δywp1* cells fail to remain adhered over time. This time-lapse darkfield microscopy movie shows the attachment of *Δywp1* cells to the substrate during the attachment phase (time indicated in the upper left hand corner; images acquired every 2 m), followed by the growth phase (starts at 2h; images acquired every 15 m). Cell-seeded media (1×10^6 cells/ml) was used during the attachment phase, while cell-free media was used during the growth phase. Flow is from the right to left. Scale bar indicates 50 µm.
